# Citizens, doctors, politicians - who´s an expert in times of COVID-19? A survey in Austria and Germany

**DOI:** 10.1186/s13690-021-00666-5

**Published:** 2021-08-16

**Authors:** Dagmar Schaffler-Schaden, Juergen Herfert, James O´Brien, Tim Johansson, Alexander Seymer, Stephan Ludwig, Thomas Stöggl, Juergen Osterbrink, Maria Flamm, Antje van der Zee-Neuen

**Affiliations:** 1 Institute of General Practice, Family Medicine and Preventive Medicine, Centre for Public Health and Healthcare Research, Paracelsus Medical University, Strubergasse 21, 5020 Salzburg, Austria; 2 Red Bull Athlete Performance Centre, Brunnbachweg 71, 5303 Thalgau, Austria; 3 The Australian Centre for Research into Injury in Sport and its Prevention (ACRISP), 6027 Joondalup, WA Australia; 4 Department of Sociology, Paris Lodron University of Salzburg, Rudolfskai 42, 5020 Salzburg, Austria; 5 Institute of Virology, University of Muenster, Von Esmarch-Strasse 56, 48149 Muenster, Germany; 6 Department of Sport and Exercise Science, Paris Lodron University of Salzburg, Schlossallee 49, 5400 Hallein/Rif, Austria; 7 Brooks College of Health, University of North Florida, Building 39, 1 UNF Drive, 32224 Jacksonville, FL USA; 8 Institute of Nursing Science & Practice, Centre for Public Health and Healthcare Research, Paracelsus Medical University, Strubergasse 21, 5020 Salzburg, Austria

**Keywords:** health experts-COVID-public information-transmission

## Abstract

**Background:**

This study aimed to explore which measures and risk factors for a COVID − 19 infection are considered most important in the general population, health experts and policymakers and to assess the level of agreement across the groups from Austria and Germany.

**Methods:**

A two-phased survey was conducted, participants were matched according to age and gender. Three different groups were asked which measures they considered most relevant in reducing a COVID-19 transmission, to determine which factors contribute most to the risk of disease, and to evaluate the level of agreement in the assessment of risk factor relevance for (a) the transmission of the disease and (b) the risk of a severe course of COVID-19.

**Results:**

Risk factors for an infection that were selected from all three groups were immunosuppression/deficiency, cancer, chronic lung disease, smoking, age and working as a health care professional. Interrater agreement per population was only poor to slight and results were highly heterogeneous.

**Conclusions:**

Our survey shows a broad spectrum of opinions and the associated general uncertainty about the risk factors for infection and a severe course of disease across the groups. Profound knowledge of politicians and experts is of high relevance to provide the public with valid information to ensure cooperation fighting the pandemic.

**Trial registration:**

https://apps.who.int/trialsearch/ (ID: DRKS00022166). Registered 15 June 2020.

## Background

The ongoing COVID-19 pandemic is particularly challenging for those providing care and guidance to the general population. The body of knowledge regarding the mechanisms of transmission and the biological pathways is increasing daily rendering it nearly impossible to make decisions based on the most recent and best evidence available. The information provided by governments and health care providers can cause confusion for both the general public and health care experts alike, particularly when confronted with contradictory information from other sources. Therefore, adherence to governmental and medical guidelines may be limited posing an additional risk in the transmission chain. Even for experts, the vast amount of available information can be difficult to navigate.

Uncertainties also exist about the presumed risk factors concerning the infection and the factors determining the severity of the disease. Published case series have identified several potential COVID-19 risk factors, including age, male gender, cardiovascular or respiratory disease, immunosuppression, and others [1–3]. Other possible factors may also be discovered in the future. It is assumed that the primary transmission happens via droplet aerosols from human-to-human but other ways of contracting the virus have been also considered possible [4].

Compared to other European countries, Austria and Germany have so far been quite successful in managing the pandemic, with intensive care units and public hospitals not reaching full capacity as yet, however, infection cases were rising rapidly in the winter season 2020/21 [5].

Expert knowledge and attitudes about infection are essential to control the spread of the disease, but to ensure that the findings are implemented in daily life, policymakers and the general population must also be adequately informed [6]. We conducted this study aiming at the exploration of the state of knowledge regarding COVID-19 in different groups of the population. More specifically, we aimed to explore which strategies were considered most effective in reducing the risk of COVID-19 transmission and which factors contributed most to the risk of COVID-19 infection according to the participants.

## Methods

In June 2020, a survey with two phases was conducted after inviting three groups of participants from Germany and Austria with various professional backgrounds. The first group included people with professional expertise in the health care sector, the second group comprised political decision-makers and the third group included participants out of the general population with neither political nor medical background. The group including persons with professional expertise was selected based on consensus among the authors. To each participant in the group with professional expertise a participant from the general and the politically active population was matched based on age and gender. The survey was designed to (1) investigate which measures were considered most relevant in reducing a COVID-19 transmission, (2) determine which factors contribute most to the risk of disease, and (3) evaluate the level of agreement in the assessment of risk factor relevance for a) the transmission of the disease and b) the risk of a severe course of COVID-19.

We invited all participants to share their opinions by filling out two short surveys received per mail. The first phase of the survey consisted of a mix of one open question and four multiple-choice questions. The open question asked participants to name one strategy they thought was most effective in reducing the risk of transmission of COVID-19. The multiple-choice questions addressed a list of risk factors for the infection that was determined by a rapid review of the literature [1, 3, 7–14]. Risk factors were categorized into four topics:

1) Comorbidities (i.e. chronic kidney, lung, liver, or cardiovascular disease, immunosuppression/immunodeficiency, cancer, diabetes, cerebrovascular disease, others).

2) Factors related to (health) behavior (i.e. smoking, alcohol consumption, consumption of pain medication or medication to reduce hypertension, others).

3) Biological determinants (i.e. age ≥ 65 years, male gender, obesity (BMI > 30), Asian ethnicity, other ethnicities, pregnancy, others).

4) Life/work conditions (i.e. working as a health care professional, increasing number of persons per household, living in a nursing home, lower socioeconomic status, living in an urban area, living in a rural area, others).

All participants were requested to indicate, using checkboxes, which factors they considered relevant. Multiple answers were permitted.

During the second phase of the survey, the participants received an overview of ten risk factors that had been selected most frequently during the first phase of the survey. They were asked to rank these factors from one (factor with highest relevance) to ten (factor with lowest relevance) based on their relevance for the transmission of the infection and the risk of a severe course of the disease, respectively (see Table 1). Furthermore, participants from the general and politically active population were asked to reveal their main source of information during the COVID-19 pandemic.

**Table 1:** example of a completed table of the second phase of survey with the ranking from 1–10

	Risk factors	A Relevance for the transmission of disease (1–10)	B Relevance for the risk of a severe course of disease (1–10)
a	**obesity (BMI ≥ 30)**	7	8
b	**age > 65**	8	10
c	**chron. cardiovascular disease**	6	9
d	**chron. lung disease**	3	4
e	**diabetes**	5	6
f	**immunodeficiency/immunosuppression**	4	3
g	**cancer**	2	5
h	**low socioeconomic status**	10	7
i	**smoking**	1	2
j	**working as a health care professional**	9	1

### Statistical analyses

All statistical analyses were performed using STATA 12.0. Strategies suggested by responses to the first question of phase 1 were listed and frequencies of answers were described. Moreover, frequencies of selected risk factors were described after phase one. To determine the level of agreement in ranks provided by the second phase of the survey, Fleiss Kappa was calculated for each rank and in total. Additional agreement statistics including percent agreement, Brennan and Prediger, Gwet’s agreement coefficient and Krippendorff’s Alpha were employed to address potential over- or underestimation of agreement attributable to the small sample under investigation. For the calculation of all agreement statistics the STATA extension “Kappaetc” by Klein was used including reference values for interpretation of the level of agreement (i.e. values < 0.0001: poor agreement, 0.0000-0.2000: slight agreement, 0.2000–0.4000: fair agreement, 0.4000–0.6000: moderate agreement, 0.6000–0.8000 substantial agreement, 0.8000-1.0000: almost perfect agreement).

## Results

After successful matching, each of the three groups of participants consisted of two women and eight men. Six were between 35 and 45 and four between 55 and 65 years of age, three resided in Germany, and 7 in Austria. Health care experts had different professional backgrounds including virology (*n* = 2), microbiology (*n* = 1), laboratory medicine (*n* = 1), nursing science (*n* = 2), public health and sociology (*n* = 1), blood analysis and diagnostics (*n* = 1), sports medicine (*n* = 1), anesthetics and intensive care (*n* = 1), healthcare management (*n* = 1) and general medicine (*n* = 1). All experts (*n* = 10) responded to both phases of the survey. Two participants provided more than one answer to the first (open) question of phase one.

Regarding the risk of infection in the first phase of the survey, health care professionals considered immunosuppression/deficiency, cancer, age > 65, and working as a healthcare professional most relevant. Participants from the general population considered age, immunosuppression/immunodeficiency, and chronic lung disease as the most relevant risk factors during the first phase, which was quite comparable to the group of the politicians.

Risk factors that were most frequently selected from the list of risk factors in phase one and therefore were selected for phase two are provided in Table 2.

**Table 2:** risk factors most frequently selected in phase 1 and taken forward to ranking in phase 2

risk factor	frequency of selection, n			agreement
	***population with healthcare background (n = 10)***	***general population (n = 7)***	***politically active population (n = 8)***	
***comorbidities***				
chronic lung disease	x	x	x	1.00
chronic heart disease	x	x		0.67
immunosuppression/immunodeficiency	x	x	x	1.00
cancer	x	x	x	1.00
diabetes	x			0.33
***behaviour***				
smoking	x	x	x	1.00
use of hypertension medication			x	0.33
***biological components***				
age > 65 years	x	x	x	1.00
male gender		x		0.33
obesity	x			0.33
***living/working conditions***				
working as health care professional	x	x	x	1.00
number of persons per household			x	0.33
living in a retirement home		x	x	0.67
socioeconomic status	x			0.33
living in an urban area		x	x	0.67

Risk factors that were selected from all three groups for phase two were: immunosuppression/deficiency, cancer, chronic lung disease, smoking, age, and working as a health care professional

No significant agreement per individual population was apparent in the ranking of the risk factors related to the risk of infection or the risk factors related to a clinically relevant course of the disease, respectively. Overall, interrater agreement per population was only poor to slight for both themes (see Table 3).

**Table 3:** interrater- agreement per population in ranking of risk factors according to their relevance for transmission and clinically relevant course of disease

	*relevance for transmission*			*relevance for course of disease*		
	coefficient	95 % confidence interval	agreement	coefficient	benchmark interval	agreement
***population with healthcare background***						
percent agreement	**0.10**	**0.06–0.14**	*slight*	**0.19**	**0.14–0.24**	*slight*
Brennan & Prediger	**0.00**	**-0.04-0.04**	*poor*	**0.10**	**0.04–0.16**	*slight*
Cohen’s Kappa	**0.00**	**-0.04-0.04**	*poor*	**0.10**	**0.04–0.16**	*slight*
Scott/Fleiss’ Kappa	**0.00**	**-0.04-0.04**	*poor*	**0.10**	**0.04–0.16**	*slight*
Gwet’s Kappa	**0.00**	**-0.04-0.04**	*poor*	**0.10**	**0.04–0.16**	*slight*
Krippendorff’s Alpha	**0.01**	**-0.03-0.05**	*slight*	**0.11**	**0.05–0.17**	*slight*
***general population***						
percent agreement	0.16	0.06–0.25	*slight*	0.12	0.07–0.18	*slight*
Brennan & Prediger	0.06	-0.04-0.17	*slight*	0.03	-0.04-0.09	*slight*
Cohen’s Kappa	0.05	-0.03-0.14	*slight*	0.03	-0.03-0.08	*slight*
Scott/Fleiss’ Kappa	0.05	0.03–0.14	*slight*	0.02	-0.04-0.08	*slight*
Gwet’s Kappa	0.07	-0.04-0.17	*slight*	0.03	-0.04-0.09	*slight*
Krippendorff’s Alpha	0.07	-0.02-0.15	*slight*	0.03	-0.02-0.09	*slight*
***politically active population***						
percent agreement	0.11	0.07–0.15	*slight*	0.14	0.10–0.19	*slight*
Brennan & Prediger	0.01	-0.03-0.06	*slight*	0.05	-0.00-0.10	*slight*
Cohen’s Kappa	0.01	-0.03-0.06	*slight*	0.05	-0.00-0.10	*slight*
Scott/Fleiss’ Kappa	0.01	-0.03-0.06	*slight*	0.05	-0.00-0.10	*slight*
Gwet’s Kappa	0.01	-0.03-0.06	*slight*	0.05	-0.00-0.10	*slight*
Krippendorff’s Alpha	0.03	-0.02-0.07	*slight*	0.06	0.01–0.11	*slight*

*benchmark-scale: <0.00àpoor, 0.00-0.20àslight,0.20–0.40àfair, 0.40–0.60àmoderate, *

*0.60–0.80àsubstantial, 0.80-1.00àalmost perfect*

Strategies considered most effective in reducing the risk of transmission of COVID-19 (open question) included:

Among health experts: contact tracing (*n* = 1), physical distance (*n* = 5), wearing face masks (*n* = 2), conducting regular PCR-tests in medical workers (*n* = 1), maintaining closure of boarders for non-essential travel (*n* = 1), and employment of specific prevention strategies for persons ≥ 70 years. Among politically active participants: employment of a contact-tracing app (*n* = 2), improvement of resources for the development of therapeutic measures (*n* = 1), an increase of capacities for the conduction of PCR-tests (*n* = 1), reduction of social contacts (*n* = 1), wearing face masks (*n* = 4) and physical distance (*n* = 1)

Among the general population: reduction of social contacts (*n* = 1), wearing face masks (*n* = 4), avoidance of gatherings with direct contact (*n* = 1), the prohibition of events in closed rooms with more than 50 persons (*n* = 1), physical distance (*n* = 2) and strengthening of the immune system (*n* = 1). As can be seen, the results of the individual groups regarding effective measures against virus transmission are highly heterogeneous with no substantial agreement across groups either. Wearing face masks was most often mentioned across the groups

Additionally, politicians and members of the general population were asked to reveal their main source of information concerning the pandemic. 30 % of both groups stated the internet as their main source. Other sources mentioned were social media, mass media (TV, newspapers), and public health organizations (WHO, Robert- Koch- Institute, RKI).

## Discussion

Our survey reveals a very broad range of opinions and associated general uncertainty regarding the risk factors for infection with the SARS- COV-2 virus and a severe course of the disease. Although several parameters were considered important from all three groups, the heterogeneity in the ranking of previously selected parameters within the populations is remarkable. Specifically, health care professionals seemed to disagree regarding the risk factors most relevant for the risk of transmission. Considering that healthcare professionals are most likely more health literate than the other two populations in our investigation, this result is rather surprising. Possible explanations may be found in the lack of conclusive evidence on risk factors for COVID-19. In their study protocol, Dzinamarira et al. have described this problem in more detail and propose a living systematic review and meta-analysis[15]. However, this information has yet to become available and at the time of data collection for the current study, the overall body of knowledge on risk factors for COVID-19 was still very limited. Despite the limited evidence, all health experts in our survey considered working as a health professional a very relevant risk factor for a COVID − 19 infection. This seems plausible since a systematic review of literature (until May 2020) on COVID-19 infection and mortality rates among healthcare workers showed that healthcare workers in Europe had the highest number of infections and deaths [16].

Regarding effective strategies to prevent the spread of the virus, there was little agreement within and among the groups which is likely attributable to the exploratory nature of the phase in the COVID-19 pandemic at the time of data collection. To date, prevention strategies across countries and regions are still incongruent and expert advice is largely based on the constellation of outbreak management teams and involved (medical) specialisms. The latter is also relevant for our own investigation: The constellation of the group of healthcare workers might have affected the reported information (i.e. there is a chance that a group consisting of individuals with different background would have provided responses that are more consistent). Yet, the heterogeneity in opinions found in our study is in agreement with other investigations in the field. Findings from different countries have been published on the subject of knowledge, attitudes, and practice with widely differing results [17–28]. In previous epidemics that affected Germany (Influenza 2009, Ebola 2014), it was already apparent that a large proportion of the population did not follow the recommendations of scientific experts. Thus, many people overestimated or underestimated their risk of infection and misperceptions about transmission routes prevailed [29]. In the Influenza pandemic 2009, risk perception was low even during the peak of the pandemic, which influenced peoples` vaccination decision [30].

Cultural and socioeconomic aspects may have also influenced opinions in our study, the perception of and access to information, and adherence to measures. However, previous study results regarding attitudes and knowledge are conflicting: While some authors report that people with poor knowledge are more relaxed about fighting the pandemic, others confess that higher levels of knowledge are associated with better confidence and positive attitudes towards the battle against the pandemic [18, 22, 24, 31]. Certainly, better knowledge is helpful to follow preventive measures [21]. A recent review including 7 studies about knowledge, attitude, and practice (KAP) concerning COVID − 19 revealed that knowledge has a direct influence on attitude and practice towards the infection [32]. Assuming that healthcare professionals are more knowledgeable in the context of the current pandemic, this may partially explain the lack of agreement on relevant risk factors between them and the other two groups in our study. Differences within the healthcare professional group may also be explained through the fact that consensus reports concerning COVID − 19 are published nearly daily but usually refer to specific comorbidities or populations, e.g. rheumatic diseases or pregnant women [33–35].

Results regarding risks factors for a COVID − 19 infection and a severe course of the disease of experts and politicians had only poor concordance in our study. Although 30 % of our study population stated to obtain their information from the internet and received quick and much information concerning the pandemic, there was low agreement. Democratic policy-making does not necessarily match decision-making based on evidence from the scientific literature. Other factors like political power relations may influence decisions [36]. However, people tend to change their behavior most in response to government action as earlier studies have shown [29]. This underlines the importance of reliable information published by health authorities who work closely together with scientific experts. Since experts and policymakers have a major influence on the opinion and behavior of the general population about the disease, their assessment appears to be of great importance.

From the present point of view, it can be assumed that with the ongoing duration of the pandemic the knowledge about the disease and its transmission will increase. Nevertheless, it seems worrying that in some countries even health care professionals appear to lack knowledge about the COVID- 19 infection [17]. To date, physical distancing seems to be the most relevant factor to prevent the spreading of the virus [37]. This parameter was also most-rated by the health professionals in our survey. However, there is also considerable controversy about how great the distance should be [7, 38]. A recent systematic review suggested physical distancing of 1 m or more as the most protective factor. A similar debate has grown about protection measures: real evidence of the protective impact of face masks and eye protection remains unclear [7].

It seems plausible that mass media and especially social media play an important role in shaping the public opinion. Young people often get information from social media or the internet [25]. Various social networks and apps provide news 24 h and also allow information to be shared quickly with many people. In our survey, the internet was mentioned as a main source of information as well, whereas social media held rather a subordinate position. This may be due to the overall higher age of the participants. The internet (and social media) provides a useful source for quick retrieval of information. However, it not only removes barriers for the access to valid information but also facilitates the dissemination of rumors and misleading news [39]. Several studies have addressed this issue and found that information concerning COVID − 19 is often consumed through the internet and social media but is not necessarily perceived as trustworthy [19, 39]. This might explain, why agreement on information on COVID-19 risk factors did not become apparent in our study: In absence of trustworthy information, people might base their decisions on self-estimation or information from their peers or family.

The so-called “infodemic”, a mix of too much and misleading information can confuse the population. Fact checking of social media and banning advertisements of medical devices and drugs that have not proven their efficacy could help to increase the confidence of the public and agreement on relevant risk factors [40].

The relevance of consistent information to the general population and education was emphasized in several studies [18]. Uncertainty also causes populations to partially disregard the measures decreed by the governments and leads to the formation of increasingly large protest movements, as the adherence to measures is significantly dependent on knowledge and attitudes [20].

Therefore, it seems of major interest for the general population to obtain accessible and authentic information from public health authorities. This is even more crucial in countries where many people cannot write or read [41]. Social media is a helpful tool for making health issues accessible to a broad public. In this context, an agreement between public health experts and politicians seems of the utmost importance. This necessitates an ongoing interaction within and between these two groups to generate comprehensive and matching information that is logically understandable for the general population.

Strengths and limitations: A limitation of this study is the small study population. Due to the small sample size and the regional data collected, the KAP might not be representable for the whole population in Austria or Germany. Since we wanted to generate comparable groups, the number of politicians and health professionals willing to participate was a limiting factor. The different professional backgrounds of the participants, who came from two countries, is an asset. However, it was not possible to collect data about education from all participants due to the wish for anonymity. Considering that education is associated with disease knowledge [21, 42], the lack of information might have affected the interpretation of results. Yet, as described above, large variation in KAP is common throughout the literature and the study reflects the general uncertainty and the wide range of opinions across the population well. Moreover, personal contact with respondents ensured complete and high-quality answers.

## Conclusions

As we demonstrated with our survey, knowledge about COVID 19 is very heterogeneous and there is practically no agreement regarding the relevance of specified risk factors of transmission and severe disease among participants from Austria and Germany. Knowledge about COVID-19 at the time of data collection was low compared to the current situation which might have contributed to the great heterogeneity in opinions. The effectiveness of measures to keep a pandemic under control is highly dependent on the knowledge and cooperation of the population and the trust in health authorities. Public awareness and individual adherence to measures are essential parameters to guarantee a successful control of the infection. It is crucial that health organizations and policymakers work closely together with experts and get reliable scientific information. The media should check scientific facts more carefully to prevent the spread of rumours and misinformation. With virtually everyone now holding a smartphone, mass media and social media could help to spread public health information quickly among the population.

## Data Availability

Data are available from the corresponding author upon request.

## Abbreviations

BMI: Body Mass Index
WHO: World Health Organization
RKI: Robert Koch Institute, Germany
COVID: Corona Virus Disease
SARS- COV-2: Severe Acute Respiratory Syndrome Coronavirus Type 2
KAP: Knowledge, Attitude, Practice
